# Author Correction: Double-negative metamaterial square enclosed Q.S.S.R For microwave sensing application in S-band with high sensitivity and Q-factor

**DOI:** 10.1038/s41598-023-35422-y

**Published:** 2023-05-22

**Authors:** Muhammad Amir Khalil, Wong Hin Yong, Mohammad Tariqul Islam, Ahasanul Hoque, Md. Shabiul Islam, Cham Chin leei, Mohamed S. Soliman

**Affiliations:** 1 Faculty of Engineering (F.O.E.), Multimedia University (MMU), 63100 Cyberjaya, Selangor Malaysia; 2 Department of Electrical, Electronic and Systems Engineering, Faculty of Engineering and Built Environment, University Kebangsaan Malaysia, 43600 Bangi, Malaysia; 3 Institute of Climate Change, University Kebangsaan Malaysia, 43600 Bangi, Malaysia; 4 Department of Electrical Engineering, College of Engineering, Taif University, P.O. Box 11099, Taif, 21944 Saudi Arabia

Correction to: *Scientific Reports* 10.1038/s41598-023-34514-z, published online 05 May 2023

The original version of this Article contained an error in Affiliation 4, which was incorrectly given as ‘Department of Electrical Engineering, Faculty of Engineering, Taif University, Taif, 21944, Saudi Arabia’. The correct affiliation is listed below:

Department of Electrical Engineering, College of Engineering, Taif University, P.O. Box 11099, Taif 21944, Saudi Arabia.

The original Article has been corrected.

